# Using antibody directed phototherapy to target oesophageal adenocarcinoma with heterogeneous HER2 expression

**DOI:** 10.18632/oncotarget.25159

**Published:** 2018-05-01

**Authors:** Hayley Pye, Mohammed Adil Butt, Laura Funnell, Halla W. Reinert, Ignazio Puccio, Saif U. Rehman Khan, Savvas Saouros, Jared S. Marklew, Ioanna Stamati, Maryam Qurashi, Rehan Haidry, Vinay Sehgal, Dahmane Oukrif, Michael Gandy, Hayley C. Whitaker, Manuel Rodriguez-Justo, Marco Novelli, Rifat Hamoudi, Gokhan Yahioglu, Mahendra P. Deonarain, Laurence B. Lovat

**Affiliations:** ^1^ Department for Tissue and Energy, Division of Surgery and Interventional Science, University College London, London, UK; ^2^ Upper Gastrointestinal Service, University College London Hospitals NHS Foundation Trust, London, UK; ^3^ Antikor BioPharma, Stevenage, UK; ^4^ Imperial College London, London, UK; ^5^ Department of Pathology, University College London, London, UK; ^6^ Sharjah Institute for Medical Research, College of Medicine, University of Sharjah, Sharjah, UAE

**Keywords:** HER2, antibody drug conjugate, photodynamic therapy, oesophageal adenocarcinoma, heterogeneity

## Abstract

Early oesophageal adenocarcinoma (OA) and pre-neoplastic dysplasia may be treated with endoscopic resection and ablative techniques such as photodynamic therapy (PDT). Though effective, discrete areas of disease may be missed leading to recurrence. PDT further suffers from the side effects of off-target photosensitivity. A tumour specific and light targeted therapeutic agent with optimised pharmacokinetics could be used to destroy residual cancerous cells left behind after resection. A small molecule antibody-photosensitizer conjugate was developed targeting human epidermal growth factor receptor 2 (HER2). This was tested in an *in vivo* mouse model of human OA using a xenograft flank model with clinically relevant low level HER2 expression and heterogeneity. *In vitro* we demonstrate selective binding of the conjugate to tumour versus normal tissue. Light dependent cytotoxicity of the phototherapy agent *in vitro* was observed. In an *in vivo* OA mouse xenograft model the phototherapy agent had desirable pharmacokinetic properties for tumour uptake and blood clearance time. PDT treatment caused tumour growth arrest in all the tumours despite the tumours having a clinically defined low/negative HER2 expression level. This new phototherapy agent shows therapeutic potential for treatment of both HER2 positive and borderline/negative OA.

## INTRODUCTION

An estimated 9,000 new cases of oesophageal adenocarcinoma (OA) are diagnosed each year in the UK and the 5-year survival is 15% [1]. OA tumours arise from the oesophagus itself, or from the junction between the oesophagus and stomach. The most important precursor for the development of OA is Barrett’s epithelium (BE), with the risk increasing as it develops dysplasia [2]. Current treatments for early stage OA and BE that is localised to the mucosal layer include endoscopic resection (EMR) and ablation. These therapies are gastroenterologist directed and despite high quality imaging, small tumours are still hard to detect and can have indistinct edges, this can lead to incomplete removal of the cancer and subsequent recurrence. Over-zealous treatment to reach deeper or suspicious but negative tumour margins can lead to normal tissue damage and oesophageal strictures or perforations [3, 4].

Photodynamic therapy (PDT) is a non-invasive therapeutic treatment for OA and can be used to treat BE with high grade dysplasia as well as advanced OA to help improve swallowing [5–7]. PDT drugs, known as photosensitizers (PS), accumulate passively in the tumour. Activation occurs via the targeted application of laser light to the tumour area. Cellular destruction occurs *via* multiple reactive oxygen species (ROS) and/or free radical pathways so consequentially resistance is rare [8–10]. PDT can also activate an immune response to cancer, a key step in establishing a prolonged remission [11, 12]. The wider acceptance of PDT has been limited by a sub-optimal pharmacokinetic profile and poor tumour selectivity, This leads to low potency and off-target photosensitivity that can cause scarring and stricture formation within the oesophagus as well as sensitivity to natural light, leading to severe ‘sunburn’ in light exposed areas [5, 13]. Antibody directed phototherapy aims to overcome this by reducing both PS clearance time and non-specific uptake [14–16]. Antibody fragments are generally taken up into the tumour more rapidly, exhibit quicker serum and tumour clearance times and generally lead to a higher tumour:normal tissue ratio than whole monoclonal antibodies [17, 18].

HER2 is an established biomarker for cancers of the digestive system [19]. The HER2-targeting antibody Trastuzumab in combination with chemotherapy is licensed for oesophagogastric cancer patients where is was shown to improve progression-free and overall survival in those overexpressing HER2 [20, 21]. However, the link between patient prognosis and HER2 overexpression remains controversial and HER2 positivity levels in the literature range from 5 to 30% in gastroesophageal junction and OA cancer patients [22–25]. This is likely due to heterogeneous expression, study bias for cancer position or grade and the previous variation in HER2 scoring across the field [26, 27].

A novel HER2 targeted phototherapeutic for PDT could be combined with existing minimally invasive endoluminal therapy to allow destruction of tumour tissue beyond localised disease and beyond that visible down the endoscope. Ideally this agent would also be available to patients with borderline or heterogeneous HER2 expression who may not have previously been offered therapies targeting HER2. C6.5 is well characterised single-chain variable fragment (ScFv) against HER2 [28, 29]. It was selected to produce a novel phototherapy agent targeted against HER2. C6.5 was re-engineered in a form optimal for bioconjugation and then reacted with a pre-activated form of the water soluble photosensitiser chlorin e6. The final phototherapy agent was tested both *in vitro* and in an *in vivo* tumour model with clinically relevant heterogeneous HER2 expression.

## RESULTS

### HER2 as a biomarker in oesophageal adenocarcinoma

**Figure 1:**
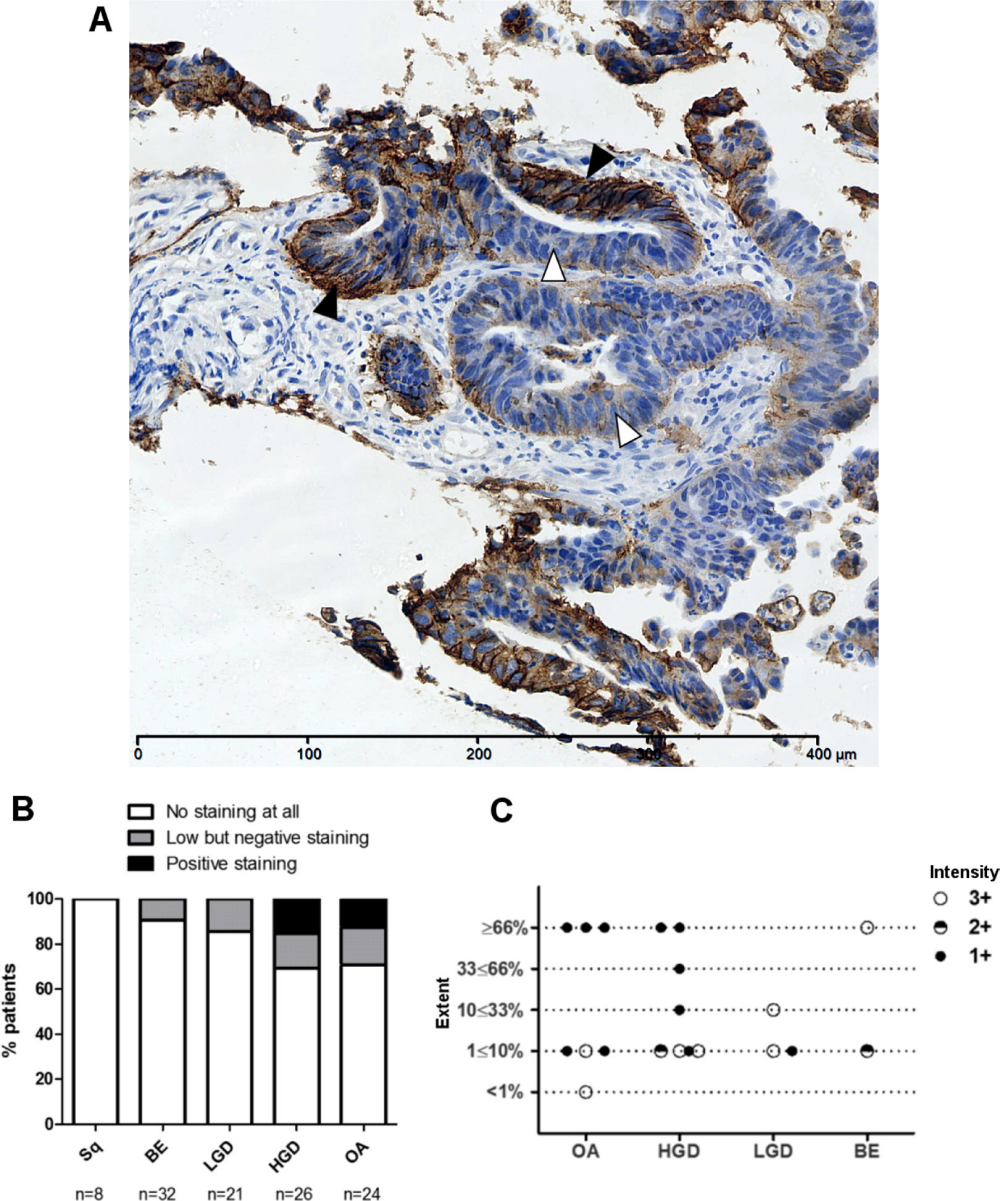
Heterogeneous HER2 immunohistochemistry in the progression to OA (**A**) An example of heterogeneous HER2 staining in HER2 positive invasive OA with areas of strong immunohistochemical HER2 positivity (black arrows), borderline staining (grey arrows) and HER2 negativity (white arrows). (**B**) HER2 expression evaluated by IHC in oesophageal adenocarcinomas and gastroesophageal junction adenocarcinomas (GOJ) as per EMEA guidelines. HER2 positivity defined as immunohistochemistry (IHC) scores of 3+ or 2+ with confirmatory in-situ hybridisation (ISH). ISH positivity defined as HER2:CEP17 ratio of ≥2; ratio <2 defining negative ISH. Borderline negative cases were categorised as those showing some HER2 staining but not sufficient to score positively. (**C**) Inset table detailing distribution of HER2 staining in oesophageal and gastroesophageal adenocarcinomas, with 2+ IHC cases subdivided by ISH status.

To confirm HER2 as a biomarker for OA, a panel of 83 oesophageal cancers originating from the oesophagus or oesophagogastric junction underwent evaluation for HER2 status. Overall, 22% (18/83) of tumours were defined as HER2 positive according to EMEA guidelines for gastric cancer [21]. The proportion of HER2 positive tumours was higher in more proximal oesophageal tumours than those originating from the oesophagogastric junction (28.2% vs 15.9%) but not significantly so (Fishers exact *p* = 0.19 (Figure 1A and 1B). A further 23.1% of oesophageal and 11.4% oesophagogastric junction tumours stained weakly (1+) or were borderline negative after confirmatory ISH (Figure 1B).

### Production of the HER2 targeted phototherapy drug; TCT-Ce6

C6.5 was the antibody selected to develop the ADC, a T7 tag was added to the C terminus and the surface exposed lysine residues were re-engineered for improved affinity and retention of function with aqueous solubility after lysine residue bioconjugation (Optilink^™^ technology Antikor). Using FACS analysis it was demonstrated that the modified C6.5 fragment (TCT) could bind live HER2 positive esophageal columnar epithelial adenocarcinoma cells (OE19) as well as a known HER2 positive gastric cancer cell line (N87) (Supplementary Figure 1).

Chlorin e6 (Ce6) is ideal for bio-conjugation as it has high water solubility for a photosensitizer (PS), a high singlet oxygen quantum yield and a strong absorption ∼660 nm [30].Ce6 was pre-activated to form an anhydride ring between two carboxyl groups to prevent cross-linking upon bio-conjugation (Supplementary Figure 2). Reaction conditions were optimised for a product that maintained the best antigen binding with retained aqueous solubility. Samples of the purified antibody–drug conjugate, TCT-Ce6, were analysed by SDS-PAGE and UV-Vis spectroscopy (Supplementary Figure 3). Unconjugated Ce6 was removed by the purification procedure and the bond formed between the TCT and Ce6 was shown to be covalent (Supplementary Figure 3A). Spectroscopic analyses predicted the average drug-to-antibody ratio (DAR) was 4 (Supplementary Figure 3B). A product with a dye to antibody ratio (DAR) of 4 was produced reliably and reproducibly between independent experiments.

### Selective binding of TCT-Ce6 to HER2 positive oesophageal cells *in vitro*

**Figure 2:**
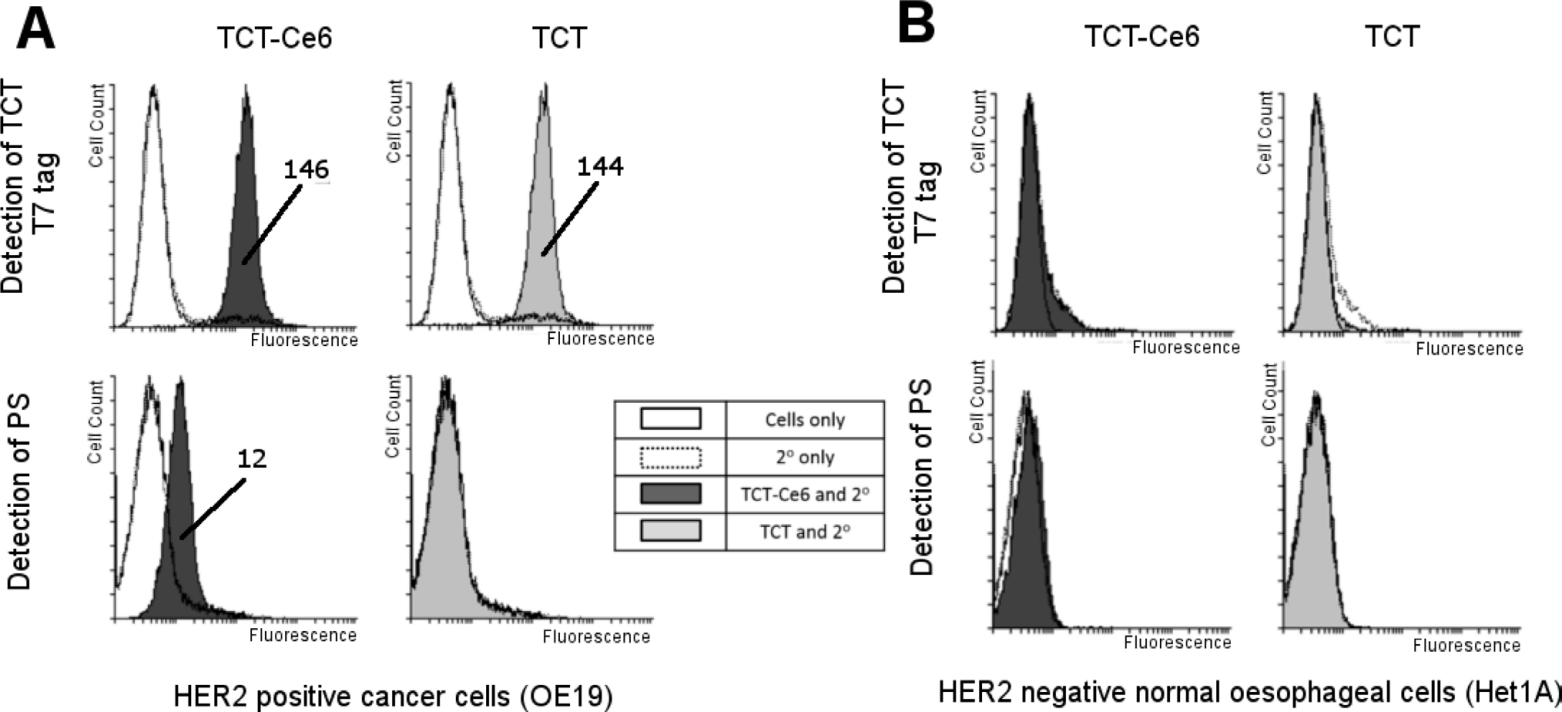
Selective binding of TCT-Ce6 to HER2 positive OA compared to HER2 negative normal esophagus *in vitro* Live cell binding of TCT-Ce6 compared to unconjugated TCT was tested on two cell lines (**A**) OE19; a human oesophageal adenocarcinoma with high HER2 expression and (**B**) Het1A; a human normal oesophageal cell line with no HER2 expression. Cell staining was carried out on ice with a previously calculated sub cell surface saturation concentration of the primary antibody TCT. This was followed by an excess of anti-T7 antibody conjugated to a fluorophore so antibody binding could be directly measured by an increase in fluorescence, cells were also measured for fluorescence from the PS dye directly. Conjugation had no effect on TCT binding to OE19 cells and Ce6 labelled cells could also be detected through PS emission. Neither TCT or TCT-Ce6 bound to Het1A cells. Geometric means of any positive shifts are labelled on the image.

Binding of the phototherapy agent TCT-Ce6 compared to the binding of unconjugated TCT was studied *in vitro* with human cell lines; the HER2 positive OA cell line OE19 and the HER2 negative normal oesophageal cell line Het1A. HER2 status of these positive and negative cells has also been shown at the RNA level (Supplementary Figure 6). No change in cell surface binding to OE19 was seen with TCT-Ce6 compared to TCT, and neither TCT or TCT-Ce6 bound Het1A (Figure 2). The cells to which TCT-Ce6 bound could also be also be detected on flow cytometry by their red fluorescent emission associated with the PS (Figure 2). Free Ce6 binding was not included in this experiment but others have shown the internalization of free Ce6 is most likely by non-specific routes including absorptive endocytosis [31].

### Light dependent cytotoxicity of TCT-Ce6 *in vitro*

**Figure 3:**
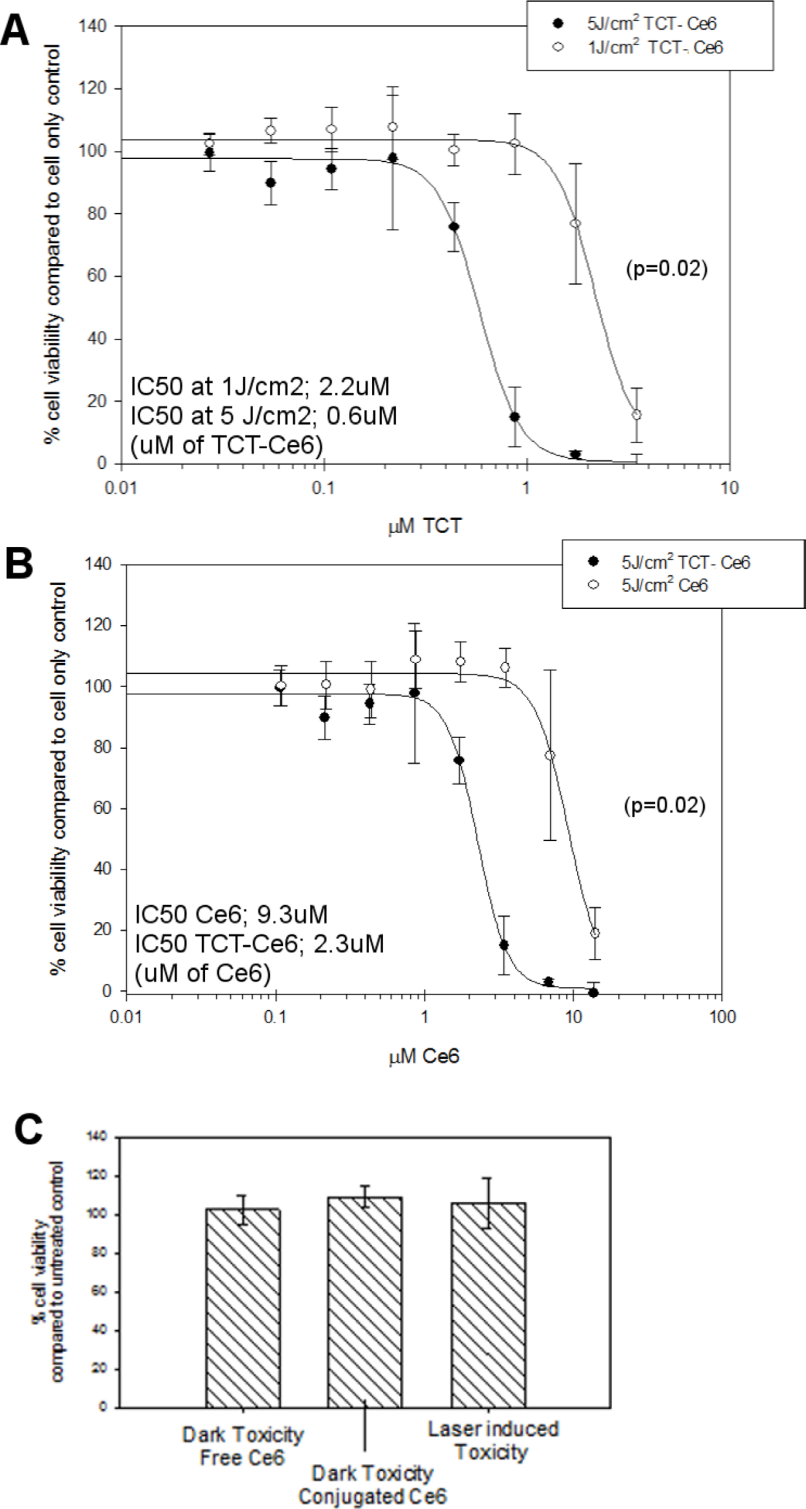
Dose and light dependent PDT cytotoxicity of the TCT-Ce6 compared to free drug on HER2 positive oesophageal cells (OE19) (**A**) The IC_50_ TCT-Ce6 at 5J/cm2 was 0.6 µΜ, with five times less light it was 2.2 µM (curves significantly different *p* = 0.02). (**B**) Comparable cytotoxicity of free Ce6 compared to conjugated Ce6. TCT-Ce6 was significantly more cytotoxic than equivalent amounts of Ce6. (**C**) Controls show no toxicity of TCT-Ce6 without light or from the laser alone. All experiments underwent the same PDT treatment; cells were exposed to various concentrations of the drug over one hour at 37° C, cells are then washed twice prior to exposing cells to a 670 nm laser. Cell viability was measured 24 hours later via MTT assay. The data shown is representative of at 6 independent repeats with various batches of drug.

To determine the activity of TCT-Ce6 for photodynamic therapy (PDT), the HER2 positive OE19 cell line was exposed to increasing concentrations of TCT-Ce6 or free Ce6 and exposed to laser light (Figure 3). Control samples showed no cytotoxicity of the drug without laser irradiation or from laser irradiation alone. Upon laser irradiation TCT-Ce6 showed light and dose dependent toxicity with an IC_50_ of 0.6 µΜ. The TCT-Ce6 induced cytotoxicity was significantly higher compared to equivalent amounts of free Ce6 (*p* = 0.02). Cells incubated at 2–4° C with TCT-Ce6 demonstrated no cytotoxicity when cells were returned to 37° C for PDT, this is consistent with previous reports that the parent antibody is internalised by endocytosis which is known to be inhibited at low temperatures (Supplementary Figure 4).

### Mouse xenograft model of human OA

A mouse flank xenograft model was developed from the HER2 positive human OA cell line OE19 subcutaneously injected into the flank of immune compromised mice (Supplementary Figure 5A). Tumours were harvested as soon as they become measurable (approx. 11 days after implantation) and at tumour burden (approx. 30 days after implantation). Tumours were stained and scored for HER2 using clinical parameters (Supplementary Figure 5B). Although *in vitro* OE19 cells exhibit high levels of HER2 expression by both flow cytometry and IHC, the *in vivo* tumours at day 11 had an average tumour area which was 81% HER2 negative, only 10% HER2 grade 1+ and 9% HER2 grade 2+ or 3+. These tumours would be classed as negative according to current NICE guidelines. There was no necrosis in these early tumours but all showed immune cell infiltrate. Tumours at day∼30 had no immune cells and demonstrated similar HER2 staining (72% HER2 negative, 19% HER2 grade 1+ and only 9% HER2 grade 2+ or 3+) alongside this an average of 30% necrosis was observed in every tumour. In all tumours at both time points 3+ staining was absent or very limited.

To confirm HER2 RNA status in the xenograft tumours two *in vivo* OE19 tumours were micro-dissected and total RNA was extracted from the areas of tumour that were HER2 positive or HER2 negative. The *xenograft* OE19 HER2 level was compared to the HER2 level in both the parental *in vitro* cell line and the *in vitro* oesophageal cell line for HER2 negativity (HET1A). Using qRT-PCR normalized to ribosomal 18S rRNA, the xenograft OE19 tumour cells showed a ∼10 fold reduction in HER2 expression compared to the parental OE19 cells (*p* < 0.00005) and a ∼20 fold increase in mRNA levels compared to the cell line Het1A (*p* < 0.00005) (Supplementary Figure 6). The difference in HER2 mRNA level between the xenograft HER2 positive and negative areas within the tumours was small but not significant in the two tumours studied.

### Pharmacokinetics of TCT-Ce6 *in vivo*

**Figure 4:**
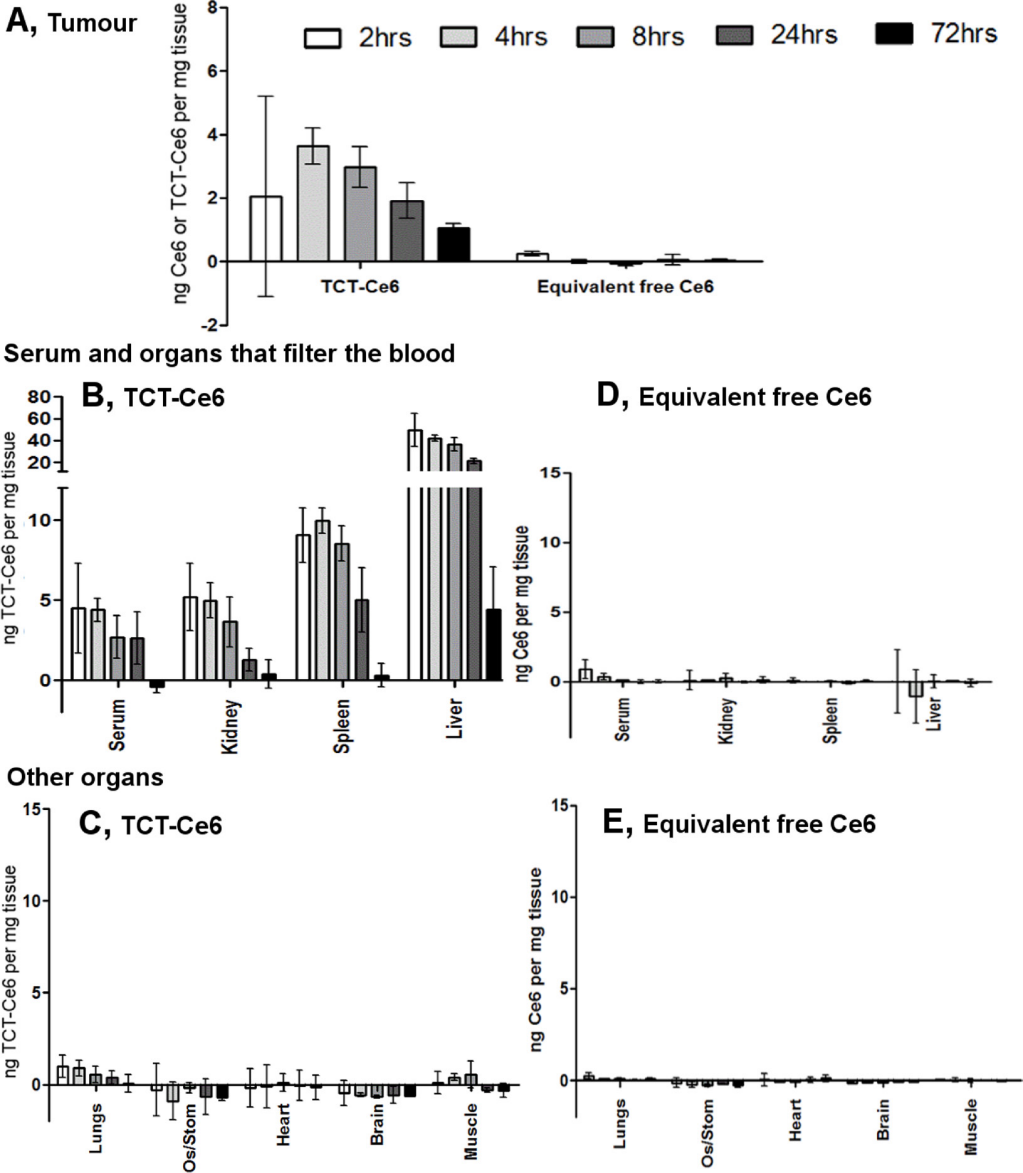
Tissue distribution of TCT-Ce6 at various timepoints after I.V injection (**A**) Distribution of TCT-Ce6 conjugate or free Ce6 into the tumour at 2, 4, 8, 24 or 72 hours after I.V injection into the tail vein (*n* = 3 at each time point). Mean data ± SEM. TCT-Ce6 accumulation in the tumour peaks at 4 hours. TCT-Ce6 (**B**, **C**) or equivalent Ce6 (**D**, **E**) distribution in the serum and other organs. Ce6 specific fluorescence measured in dissolved tissue, results controlled for tissue specific auto-fluorescence and quenching with standard curves of either free or conjugated Ce6 dissolved in each tissue (Supplementary Figure 7) as well as the lower fluorescent efficiency of the Ce6 once conjugated (∼20% of free Ce6).

The organ distribution of TCT-Ce6 or free Ce6 *in vivo*, was determined at 2, 4, 8, 24 and 72 hours after drug was injected into the tail vein of the xenograft model. Tissues from various time points were dissected and dissolved whole in a strong alkali/surfactant mixture in which the fluorescence of Ce6 or TCT-Ce6 could be directly measured. High levels of TCT-Ce6 were found in organs which filter the blood (liver, kidney, spleen), negligible levels of TCT-Ce6 was found in all other organs tested (lung, brain, heart, muscle and esophagus/stomach). TCT-Ce6 had virtually cleared the body by 72 hrs. Peak TCT-Ce6 accumulation in the tumour occurred after 4 hours (Figure 4). Equivalent amounts of free Ce6 were studied in the same model, Ce6 cleared all organs quickly and levels everywhere were negligible after 4 hrs (Figure 4). Standard curves to account for tissue specific effects and a difference in conjugated and unconjugated Ce6 fluorescence levels are shown in (Supplementary Figure 7).

Xenograft tumours and the skin directly above the tumour, were harvested 4 and 24 hours after TCT-Ce6 and stained by IHC for T7 (to detect the T7 tagged TCT-Ce6) and HER2 (Supplementary Figure 8). At 4 hours TCT-Ce6 membranous staining was seen specifically in the tumour tissue and not in the surrounding stroma or vasculature, suggesting selective uptake into the tumour (Supplementary Figure 8). At 24 hours the pattern was similar but the amount of TCT-Ce6 was negligible in many samples. There was no TCT-Ce6 in the skin above the tumour at any time point. At 4 hours TCT-Ce6 and HER2 co-localised in the same areas but TCT-Ce6 was also widely distributed throughout the HER2 negative regions of the tumour (Supplementary Figure 8).

### Effective photodynamic therapy using TCT-Ce6 *in vivo*

The *in vivo* PDT protocol was optimised in small scale experiments with increasing laser doses up to 200 J/cm^2^ and showed no laser induced skin sensitivity or blistering at any time point after drug injection (4, 8, 24, or 72 hrs). 4 hours post injection was the most effective time point which correlates with previous PK data on maximal TCT-Ce6 levels in the tumour (Figure 4).

**Figure 5:**
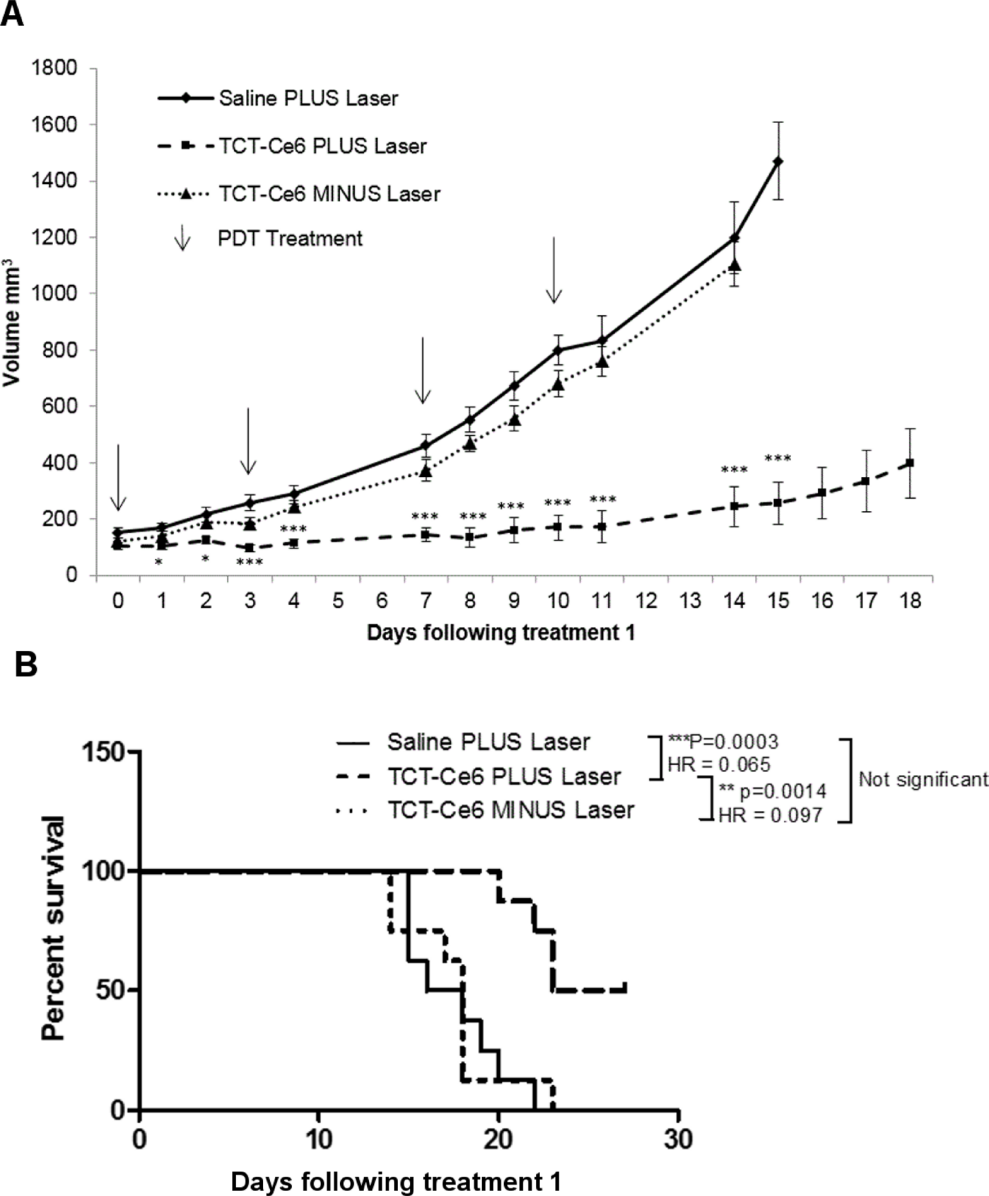
Tumour growth arrest *in vivo* after PDT treatment using TCT-Ce6 on OE19 subcutaneous flank tumours (**A**) Each group *n* = 8. Data shown as Mean ± SEM. Arrows represent individual i.v./laser treatment time points. Stars represent significant differences between Saline and TCT-Ce6 plus laser treated mice. ^*^p < 0.05, ^***^p < 0.001. PDT treatment showed significant tumour growth reduction compared to drug without laser activation and laser irradiation alone. Individual animal responses and survival curves shown in supplementary data. Kaplan Meier survival curves for each treatment group are shown (**B**) and analysed in a pairlike manner with a Log-rank (Mantel-Cox) Test (PRISM); the p value and hazard ratio (HR) for each pair is shown in the key.

OE19 xenograft tumours of equivalent volume were grown in 24 mice before treating with a single PDT treatment twice weekly for a total of 4 treatments. TCT-Ce6 phototherapy induced tumour growth arrest in all tumours treated (Supplementary Figure 9). Tumour volume was significantly different between TCT-Ce6 treated tumours with and without laser from the third day of treatment (day 3 (*p* < 0.05), day 4 (*p* < 0.01), days 5–14 (*p* < 0.001) (Figure 5). Tumour volume was significantly different between lasered tumours ± TCT-Ce6 from the second day of treatment (day 2: *p* < 0.05, days 3–15: *p* < 0.001) (Figure 5). Four of the TCT-Ce6 treated mice with laser irradiation did not reach tumour burden by the end of the study (Supplementary Figure 9). There was a significant survival benefit in mice receiving treatment (hazard ratio of 0.65 between the saline plus laser and TCT-Ce6 plus laser treatment groups (*p* = 0.0003)) (Figure 5). At the end of the study tumours were harvested and immunostained for HER2. The intensity and extent of HER2 staining was not significantly different in treated vs non-treated tumours (Supplementary Figure 9). Dark necrotic patches were observed in some tumours in the area under the skin in treated mice that had demonstrated a greater PDT effect (*n =* 4) this was sometimes seen alongside discolouration in small areas of the liver, upon histological analysis of the discoloured liver sections there was no evidence of cell damage, fat deposition, overt fibrosis or inflammation in these tissues. Some of these mice also exhibited lymph node enlargement that was not seen in the control mice.

A small study (*n =* 2) in which the same treatment regimen was tested with the equivalent dose of free Ce6 was also carried out and showed some effect (Supplementary Figure 9). The photosensitser Ce6 was chosen because of its suitability for bio-conjugation, the project was not aiming to improve free Ce6 by targeting it. Therefore it was felt that continuing with a control in which irradiation was carried out at a time that was optimal for the conjugate and not the free Ce6 was not relevant and thus an unfair comparison. Work to determine the optimal irradiation time for free Ce6 and carry out all the suitable additional controls was decided to be an excessive use of animals and not in line with the projects aims so was not done.

## DISCUSSION

HER2 targeted therapy for cancer is in routine clinical use for breast and gastric tumours [21, 32]. In both cancer types, only a subset of patients meet defined criteria for HER2 positivity, rendering HER2 directed therapies inaccessible for the rest. It has been shown that some of these HER2 negative tumours still have low or heterogeneous HER2 expression [32, 33]. In this study we defined HER2 expression in a cohort of patients with OA. We demonstrated HER2 positivity, as defined by NICE guidelines, occurs in 22% of patients with OA (Figure 1A). This is consistent with previous literature for OA and similar to the levels seen in gastric and breast cancer [25, 32, 34]. We found an additional 16.9% of OA’s were classified as HER2 negative but still demonstrated low or heterogeneous HER2 staining (Figure 1B). We believe these patients could still be targeted for HER2 therapy. Previously it has been shown that when HER2 negative gastric cancers are revaluated with either repeat endoscopic biopsy, or repeat sampling from metastatic or recurrence sites, in 8.7% and 5.7% respectively tumours were re-classified as HER2 positive [35]. In breast cancer, tumours with HER2 amplification and negative or borderline HER2 expression have been shown to have worse disease free survival compared to those with HER2 over expression [36]. Often studies examining the impact of HER2 heterogeneity in gastric cancer excluded those expressing HER2 at low levels and so their impact on prognosis is yet to be established [37, 38]. In breast cancer there is evidence that a subset of HER2-negative patients can still respond to Trastuzumab and it has been postulated that undetectable sub populations of HER2 positive cells within HER2 negative tumours are responsible for tumour growth re-initiation [39–41]. It has also been shown that histologically HER2 negative breast cancer tumours can still have increased HER2 at the mRNA level compared to normal tissue [39].

In a mouse flank xenograft model we replicated the clinical presentation of tumours with both borderline HER2 expression and intratumoural heterogeneity that would be classified as HER2 negative according to current standards (Supplementary Figure 5). IHC staining techniques and expert pathologist scoring of slices taken throughout the dissected xenograft tumours found similar HER2 expression to the borderline patient samples. Despite the low levels of HER2 expression treatment with a HER2 targeting phototherapy drug (TCT-Ce6) resulted in a rapid and significant effect on tumour volume compared to controls (Figure 5). A novel and sensitive IHC assay for the drug demonstrated that it was tumour specific and cell membrane localised in both HER2 positive and negative areas of the tumour (Supplementary Figure 8). This supports the evidence from qPCR that HER2 is still expressed in these areas. Modern sequencing techniques have demonstrated the complexity and heterogeneity of all cancers and the validity and utility of all current biomarkers are now being challenged [42, 43].

PDT is already in clinical use to treat certain aspects of OA but wider application has been limited by the sub-optimal pharmacokinetic profile and poor tumour selectivity of PS drugs [5–7, 13]. Targeting of PS with antibody directed phototherapy aims to remove these limitations. In this work a HER2 targeted PDT drug was developed. The new antibody drug conjugate (Ce6-TCT) confirmed that antibody targeting of a PS improves selective binding and cytotoxicity compared to free PS *in vitro* (Figures 2, 3). It demonstrated good *in vivo* pharmacokinetics e.g. fast serum clearance (days) compared to traditional PDT (weeks) and reduced accumulation in off target organs in particular the skin allowing repeated dosing of our drug (Figure 4 and Supplementary Figure 8). The skin is an organ in which drug accumulation in traditional PDT causes particularly bad side effects [10]. PDT has a strong immune component and the requirement of the immune system for prolonged relapse from cancer is becoming evident, *in vivo* tumours will almost always regrow after PDT unless some element of the immune system is reconstituted [15, 44–46]. Further testing of this drug in an immune competent system and further modulation in combination therapies alongside better molecular and genetic stratification of patient samples should help define a clear role for drugs like this in clinical practice.

Treatment options for oesophageal adenocarcinoma (OA) are limited and curative treatment pathways are only suitable for 30% of patients. Even after successful surgery median survival is only 15 months and relapse is common with no curative options. Photodynamic therapy is NICE approved for palliative treatment in OA but off-target toxicity limits its use. In recent years, molecular agents targeting HER2 have been added to improve survival of upper gastrointestinal cancers. The proportion of HER2 positive OA patients however is low and heterogeneity of expression limits it role as a therapeutic target. Our light-activated antibody drug conjugate has shown tumour regression in an *in vivo* animal model replicating borderline but negative HER2 heterogeneity so could offer an alternative therapeutic option for the large number of patients that are HER2 positive or borderline negative, patients with localised disease that are unfit for curative resection or patients who have relapsed after first-line therapy.

## MATERIALS AND METHODS

### Cohort

A panel of formalin fixed paraffin embedded (FFPE) oesophageal and oesophagogastric junction cancer specimens were identified from the upper gastrointestinal service at University College London Hospital. Ethical approval was obtained from the UK Research Ethics Committee (EC13.13; 08/H808/8; 08/H0714/27). Samples were selected from 83 patients, 44 with oesophagogastric junction cancer and 39 with oesophageal adenocarcinoma. Sections were stained with haematoxylin and eosin (H and E) and the reported pathological grade confirmed by a specialist gastrointestinal pathologist (MRJ or MN).

### HER2 immunohistochemistry, *in-situ* hybridization and scoring

Immunohistochemical (IHC) staining was performed on 4μm slices of paraffin-embedded tissue using the automated Bond-Max system (Leica) and a citrate based epitope retrieval solution at pH 6.0 (30 min). Endogenous peroxidase activity was blocked using 0.3% hydrogen peroxide (5 min). The primary antibody against HER2 (NCL-L-CBE-356, Leica), was diluted 1:100. Slides were incubated at room temperature with primary antibody for 15 minutes followed by secondary rabbit anti-mouse for 8 minutes and finally a tertiary goat anti-rabbit polymer reagent for 8 min. This was developed using a bond polymer refine detection kit using 3,3′-diaminobenzidine tetrahydrochloride as a chromogen over 10 min.

Samples were reported for HER2 status by an expert pathologist (MRJ or MN). Positivity requires complete, basolateral or lateral membrane staining of 3+ intensity, or 2+ intensity with confirmatory positive in-situ hybridisation in ≥10% of tumour resections specimens, or ≥5 cells with the same pattern in at least one tumour cell cluster for biopsy samples. This is in line with NICE and EMEA guidelines for HER2 testing in oesophagogastric cancer.

HER2 gene expression was evaluated in comparison to chromosome enumeration probe 17 (CEP17) using dual-colour, dual-hapten (DDISH, Ventana HER2 Dual ISH DNA probe cocktail) *in situ* hybridization for samples with 2+ intensity with the required HER2 staining pattern. 20–40 cells were evaluated and the HER2:CEP17 gene ratio calculated. Amplification ratios were scored as negative (non-amplified) (≤1.80), borderline negative (1.81-1.99), borderline positive (2.00–2.19) or positive (amplified) (≥2.20). In borderline cases, an additional 20-40 cells were reviewed. The final compiled report used HER2:CEP17 ratio ≥2.00 to define positivity.

### T7 immunohistochemistry

IHC analysis was carried out on 4μm slices of paraffin-embedded tissue. The primary antibody against T7 (ab9115, Abcam) was diluted 1:1000. Immunostaining was carried out using the automated Bond-Max system (Leica) using on board heat-induced antigen retrieval and a citrate based epitope retrieval solution at pH 6.0 (30 min). Endogenous peroxidase activity was blocked using 0.3% hydrogen peroxide (5 min). The histological specimens were incubated at room temperature with primary antibody for 30 minutes, followed by secondary rabbit anti-mouse for 16 minutes and finally a tertiary goat anti-rabbit polymer reagent for 8 min. This was developed using a bond polymer refine detection kit using 3,3′-diaminobenzidine tetrahydrochloride as a chromogen over 10 min. Samples were reported by an expert GI pathologist (MN).

### RNA extraction

For each FFPE tumour sample; A slice (6µm) of paraffin-embedded tumour tissue underwent IHC analysis for HER2, two areas were identified by microscope that were either majority HER2 negative or majority HER2 positive (any grade). The adjacent 6 slices were cut fresh from the same block, a drop of 100% EtOH was added to the freshly cut slides to help hydrate the tissue and visualise tissue structures. The slides were orientated alongside the HER2 stained slide under a microscope and a needle was used to scrape the ‘areas of interest’ into 100 ul digestion buffer (Ambion Recoverall Total Nucleic acid isolation kit), combine cells from all 6 slides into one Eppendorf (DNA LoBind), one tube per ‘area of interest’. The kit was followed as per instructions (Ambion Recoverall Total Nucleic acid isolation kit 1975MC) including the on membrane DNAse treatment step. For each cell line; one million cells of low passage at 70–80% confluent were detached from the flask with trypsin/EDTA, washed in PBS and pelleted. Dry pellets were snapfrozen in liquid nitrogen and stored at -80oC. For RNA extraction the Qiagen RNAeasy Minikit (74104) was used with on column DNAse digestion. All samples was eluted in DEPC treated water (Invitrogen 750023) and concentration and purity were assessed using the absorbance maxima at 260 and 280 nm (DS-11 FX+ Spectrophotometer, Denovix) and stored at –80° C.

### qPCR

Primers were designed that span ∼100-200bp over an exon/exon boundary for both HER2 and 18S rRNA (normalisation gene). HER2 FWD (ACATGCTCCGCCACCTCTACCA) HER2 REV (GGACCTGCCTCACTTGGTTGTG) 18SrRNA FWD (TGACTCAACACGGGAAACC) 18SrRNA REV (TCGCTCCACCAACTAAGAAC). Primer specific first strand cDNA synthesis was carried out on 200ng RNA (Superscript First-strand synthesis System for RT-PCR Invitrogen 11904-018) including a final RNAse step. (For the cell line samples an oligo dT primer was used for first strand synthesis). qPCR was carried out in triplicate for each cDNA sample for each gene of interest using SYBRGreen Supermix (Biorad 1725121) on the Biorad CFX connect Real-time system. Data was analysed using the 2^-ΔΔCt method [47]. Specificity of primers was confirmed with melt curve analysis.

### Chemical synthesis of the photosensitiser Chlorin e6-anhydride (Ce6-Anhydride)

Modified from Chen *et al.* 2015 [48] and Xu *et al* 2008 [49]. To a stirred solution of chlorin e_6_ (100 mg, 0.17 mmol) and *N*,*N*-diisopropylethylamine (58 µL, 0.15 mmol) in anhydrous DMF (2 mL) at room temperature was added HATU (57 mg, 0.15 mmol) and stirred protected from light for 1 h. The crude reaction mixture was then concentrated under reduced pressure and purified by preparative TLC eluting with anhydrous acetone and the residue recrystallised from dichloromethane with *n*-hexane. MS (ES-ToF) *m/z* 579.2 [M+H]^+^

### Production of antibody drug conjugate

C6.5, a fully human anti-HER2 single-chain Fv was first described by Schier *et al.*, 1995 [29]. The original clone from Prof J. Marks (University of California, SanFrancisco), was re-engineered, expressed and purified by Antikor Biopharma (sequence proprietary information). The new fragment, now referred to as TCT, was stored at –20° C at 12 mg/ml in sodium acetate buffer with NaCl at pH5.0, MW 28160 Da. Prior to use, TCT was filter sterilised through a 0.22 µM PVDF membrane, and diluted into PBS pH7.4 as required. For chemical synthesis of the photosensitizer Chlorin e6-anhydride (Ce6-Anhydride) see methods. Ce6-Anhydride (Antikor Biopharma) MW 579 was stored as a solid at 4° C in the dark under vacuum in a desiccator. Inactivated Ce6 (Medkoo Biosciences Cat. 500410) was stored in the dark at –20° C. Handling of the photosensitive drugs and subsequent conjugates was carried out under dim light conditions. At least 24 hours prior to reaction the Ce6-anhydride was diluted to 20 mg/mL in anhydrous DMSO, snap frozen and stored at –20 C. Frozen aliquots were used within 3 days. Conjugation reaction; for a 700 μL reaction volume; TCT was diluted to 35.5 μΜ in PBS (pH 7.4). To this the Ce6-anhydride was added (final concentration 350 μΜ) with 10 volumes of DMSO to give a 20% solution. Reaction mixture was incubated in a closed eppendorf in the dark on a flatbed shaker (125 rpm) at 37° C for 2 hours centrifuging to remove any precipitated material (10,000 g, 2 minutes) and filtered through a 0.22 um filter. Excess reagents were removed by desalting (7 kDa MWCO Zeba) into fresh buffer (PBS, pH 7.4). The average antibody recovery between batches was ∼70%. The product was stable for up to a month at 4° C in PBS pH7.4. In addition, snap frozen TCT-Ce6 stored at –20° C demonstrated equivalent cell binding and spectroscopic properties once thawed.

### SDS PAGE

Samples diluted into pH6.8 Tris-HCl loading buffer with final concentration of 2% SDS and 10% glycerol without reducing agents or tracking dyes and boiled for 5 minutes at 70–100° C. Gels were hand cast 1 mm gels with a discontinuous buffer system containing 0.1% APS and 0.1% TEMED to polymerise; Resolving gel: 12% acrylamide/bis 0.1% SDS, in 0.37 M Tris-HCl pH8.8. Stacking gel: 4% acrylamide/bis 0.1% SDS, in 0.12 M Tris-HCl pH6.8. Samples were loaded at 2 μg protein alongside a marker (Thermo 26619). Gels ran at 30 mA per gel until bands well resolved in 1X running buffer (0.25 M Trisma base + 2.5 M glycine + 0.1% SDS). Un-stained gels were imaged for fluorescence using a CCD camera flat bed imager using a Blue light LED tranilluminator (Ex450-485 nm Em >500 nm) (G:BOX CHEMI HR1.4, Syngene). Gels were then fixed and stained with 0.1% Coomassie Brilliant Blue G in a 10% acetic acid 40% methanol buffer.

### UV-Vis spectroscopy

Read using a Lambda 25 (Perkin Elmer) in a micro volume 1 cm path length quartz cuvette, samples diluted 1:20 into PBS (pH7.4). Spectra normalised to 900 nm and solvent background removed. Concentrations were calculated using the following equation A = εlc where A is absorbance of the sample, ε = molar absorptivity, l = path length in cm and c = concentration in molar. Molar extinction coefficient (M-1 cm-1) were calculated in PBS pH 7.4 as follows; TCT 280 nm ε = 65235, Ce6-anhydride 280 nm ε = 9816, 402 nm ε = 81020, 654 nm ε = 17353.

### Bradford assay

Samples were diluted 1:25 into Bradford reagent (Sigma B6916) in a 96 well plate, shaken (350 rpm) for 5–10 minutes at room temperature then measured at 630 nm on a ELx800 Absorbance Microplate reader (BioTek). A standard curve of BSA was included on each plate and read simultaneously. BSA and TCT were shown to create identical standard curves and samples were loaded at a concentration below which Ce6 would contribute to absorbance at 630 nm within the assay, previously calculated (0.6 mM).

### Cell culture

The HER2 positive oesophageal adenocarcinoma cell line OE19, the gastric adenocarcinoma cell line N87 and the HER2 negative immortalised normal squamous epithelial oesophageal cell line Het1A were obtained directly from the European Collection of Authenticated Cell Cultures (ECACC) or the American Type Culture Collection (ATCC) = OE19 (ECACC 96071721 MAY 2014) N87 (ATCC CRL-5822 NOV 2013) and Het1A (ATCC CRL-2692 OCT 2014) and cultured according to their recommendations. Cells were grown and frozen down in batches and each batch was confirmed mycoplasma free by testing of one thawed vial per group with LookOut Mycoplasma PCR Detection Kit (SIGMA MP0035). All experiments were carried out with cells kept within a 30 passage range of cell line acquisition.

### Flow cytometry

Cells were detached with Accutase (millipore SCR005), and 200,000 cells per sample were washed and incubated on ice with various concentrations of TCT. After 1 hour cells were washed and incubated with 300 nM rabbit α-T7 Tag IgG DyLight488 conjugate (Abcam ab117486) on ice for 30 minutes before two final washes. All steps carried out in FC buffer (PBS + 2% FCS + 1 mM EDTA). Flow cytometry was carried out on a Beckman-Coulter Cyan ADP, FITC detection channel; (Ex 488 nm Em 510–550 nm), PS detection channel; (Ex 635 nm Em655–675). Data from 10,000 cells was gated to exclude, doublets, aggregates and debris. Single colour controls were used to ensure there was no bleed-through between the detection wavelengths. Data was analysed and quantified using the geometric mean of the curve using Flowing Software Version 2.5.1 (Perttu Terho, Turku Centre for Biotechnology, Finland). For analysis of the ADCs TCT antibodies were incubated at 30 nM, a concentration shown to be less than the cell surface saturation of OE19 cells (Supplementary Figure 1).

### *In vitro* photodynamic therapy (PDT)

25,000 OE19 cells were plated in clear bottomed black walled 96 well plates. The following day media was replaced with media containing experimental compound at varying concentrations. Plates were protected from light and incubated at 37° C and 5% CO_2_ for one hour (for low temperature experiments (Supplementary Figure 4); media was used at 4° C and the plate incubated on ice). Cells were washed twice with PBS and returned to warm media before being exposed to a 670 nm Laser (Hamamatsu LD670C) at a dose of 5 J/cm^2^ delivered at 80 mW/cm2. Non-irradiated control cells were protected from light and returned to the incubator. Light was delivered via fibre optic/frontal light distributor (model FD-1 Medlight S.A SN FD1-1345) and pre-calibrated for exact energy delivery (Gentec TDM-300 / PSV-3103). In order to access remaining cell viability 24 h later, media was replaced with MTT reagent (Sigma-Aldrich M5655) at 0.5 mg/mL in FCS free cell culture media, more specifically the MTT assay measures the reducing ability of cells, i.e cells with metabolic activity. Plates were protected from light and incubated at 37° C and 5% CO2 for two hours, MTT media was replaced with 100 μL DMSO and shaken until all crystals had dissolved. A490 nm was measured on a ELx800 Absorbance Microplate reader (BioTek). To calculate IC50 data was fitted using SigmaPlot (Systat Software Inc.) with a Four Parameter Logistic Curve according to the equation (f1 = min + (max-min)/(1 + (x/IC50)^(-Hillslope)). Statistical difference between curves was tested with area under the curve analysis according to Cleves *et al.* [50].

### *In vivo* mouse tumour model

All experiments were executed in compliance with institutional guidelines and regulations and under our Home Office Licence (M C Loizidou 70/7666). Female SCID (CB17/Icr-PrkdcSCID/IcrIcoCrl) mice were purchased from Charles River Laboratories (Margate, UK). Mice (7–10 weeks) were inoculated with 7 million OE19 cells subcutaneously (s.c.) into the depilated right dorsal flank in a volume of 0.2 ml PBS. Tumour growth was monitored at least 3 times a week using digital vernier calipers (volume = (length × width × depth)/2). Mice were sacrificed when tumour measurements exceeded 2 cm × 1.5 cm in two dimensions, if tumour volume was calculated to be higher than 2000 mm^3^ or if the animals were exhibiting any adverse effects that affected their welfare as defined in our license. Tissues were fixed immediately in 10% neutral buffered formalin and processed to formalin fixed paraffin embedded (FFPE) blocks for analysis.

### *In vivo* pharmacokinetic study

Once tumours reached a suitable size (approximately 120 mm^3^ in 2 weeks), 30 SCID mice were treated with 0.1ml of either TCT-Ce6 (1mg/ml) or the equivalent amount of free Ce6 intravenously (i.v.) into the tail vein. Mice were sacrificed at 2, 4, 8, 24 and 72 hours after injection (*n =* 3 per time point) and their tissues harvested for analysis. Control tissue was harvested from 3 tumour bearing SCID mice following i.v. injection of PBS to control for tissue specific autofluorescence and quenching. Tumours failed to grow in 3 mice, thus *n =* 2 for Ce6 treated mice sacrificed at 4, 8 and 24 hours. Tissues and serum were collected and snap frozen immediately. Thawed tissue was individually weighed and dissolved into Solvable™ (Perkin Elmer) at 37.5 mg/ml and standards made of known amounts of either TCT-Ce6 or Ce6 dissolved in control tissues at 37.5 mg/ml in Solvable™. Fluorescence of all samples was measured in black walled 96 well plates with excitation at 400 nm and emission at 660 nm. Standard curves were fitted with Y = mX + C where Y = RFU, X = ng Ce6 (either free or within the conjugate), M = gradient or quenching power and C = Y-axis intercept or autofluorescence. Standard curves were used to calculate the concentration of Ce6/mg tissue.

### *In vivo* PDT treatment

Eleven days after tumour implantation 24 SCID mice were assigned to treatment groups: Saline (*n =* 8), TCT-Ce6 plus laser (*n =* 8) and TCT-Ce6 minus laser (*n =* 8). Mice were treated with 0.2 ml of either saline or TCT-Ce6 (0.5 mg/ml) injections (i.v.). Four hours following treatment a 2 cm diameter spot covering the tumour was illuminated using a 200 J/cm2 laser dose (150 mW/cm2 over 22 min 11sec). Laser power was kept at or below 150mW/cm2 to prevent tissue heating. The rest of the mouse was covered with a black cloth to limit illumination of normal tissue, and the room was maintained in dim light. TCT-Ce6 plus laser and saline treatment groups received laser illumination, while TCT-Ce6 minus laser mice received the equivalent duration of anaesthesia only. Mice were treated twice a week for 2 weeks (days 11, 14, 18, 21 following tumour inoculation) and kept under slightly subdued-lighting conditions throughout treatment and the following day. Animals were observed and tumours measured 3 times a week until experimental end point up to 40 days after tumour inoculation. For the small study featured in (Supplementary Figure 9C) the protocol was exactly the same apart from either the equivalent amount of free Ce6 (0.1 ml 0.08 mg/ml) (*n =* 2) or 0.1ml saline (*n =* 2) was injected (i.v.) at each treatment point. One-way ANOVA with Bonferroni’s post-hoc multiple comparison tests was used to determine significant differences in tumour volume between groups using the SPSS program (IBM Corp. Armonk, NY). Kaplan-Meier survival curves were plotted and regression model multivariate analysis was carried out using Cox’s proportional hazards model (Log-rank Mantel-Cox Test) (GraphPad PRISM Software, San Diego, CA) [51].

## SUPPLEMENTARY MATERIALS FIGURES



## Abbreviations

(OA): Oesophageal adenocarcinoma
(PDT): Photodynamic therapy
(HER2): Human epidermal growth factor receptor 2
(BE): Barrett’s epithelium
(EMR): Endoscopic resection
(PS): Photosensitizers
(ROS): Reactive oxygen species
(ScFv): Single-chain variable fragment
(Ce6): Chlorin e6
(DAR): Drug-to-antibody ratio
(FFPE): Formalin fixed paraffin embedded
(IHC): Immunohistochemical
