# Supplementary material for: Using antibody directed phototherapy to target oesophageal adenocarcinoma with heterogeneous HER2 expression
Source: Oncotarget. 2018 May 1;9(33):22945–59. doi: 10.18632/oncotarget.25159 (PMC5955430; doi:10.18632/oncotarget.25159)
Supplement: Supplementary file 1 [file oncotarget-09-22945-s001.pdf]

# Using antibody directed phototherapy to target oesophageal adenocarcinoma with heterogeneous HER2 expression

## SUPPLEMENTARY MATERIALS

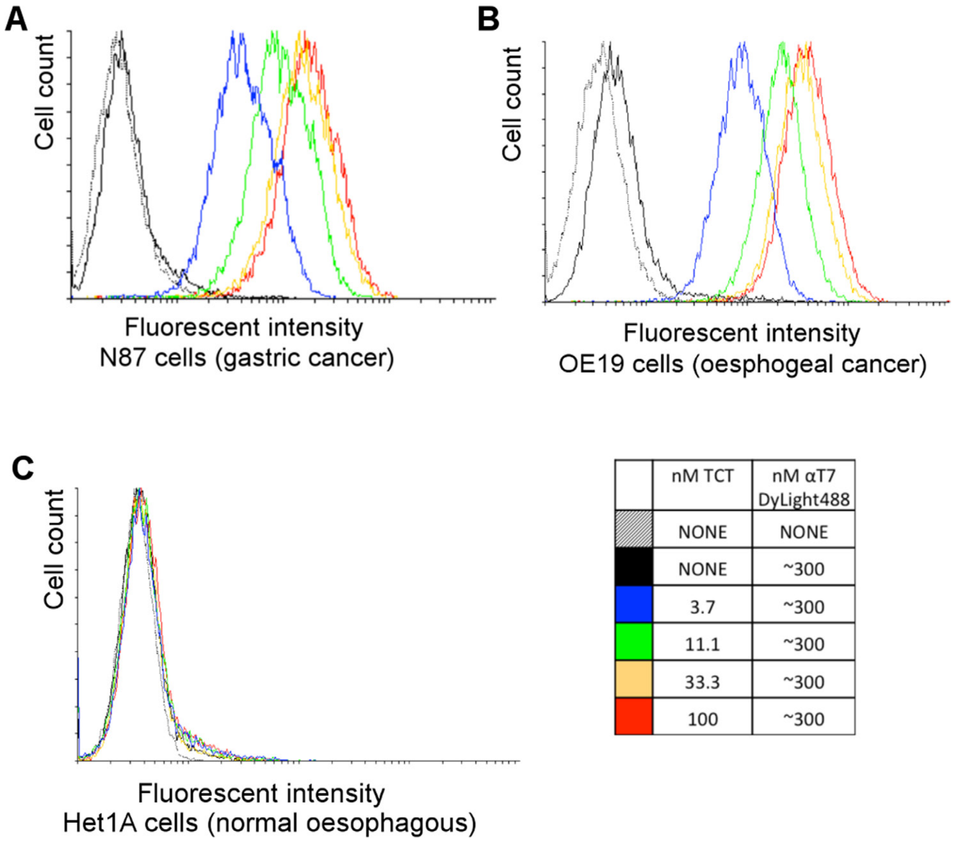


**Supplementary Figure 1: Binding of TCT to HER2 positive OA and gastric cancer *in vitro*.** Flow cytometry showing binding of the HER2 specific ScFv TCT onto (**A**) a known HER2 positive gastric cancer cell line (N87), (**B**) a HER2 positive oesophageal columnar epithelial adenocarcinoma cell line (OE19) and (**C**) a HER2 negative oesophageal squamous epithelium cell line (Het1A). Fluorescent secondary antibody used to detect the T7 within TCT, an increase in fluorescence represents more TCT has bound to each cell and the saturation of the fluorescent signal indicates cell surface receptor saturation.

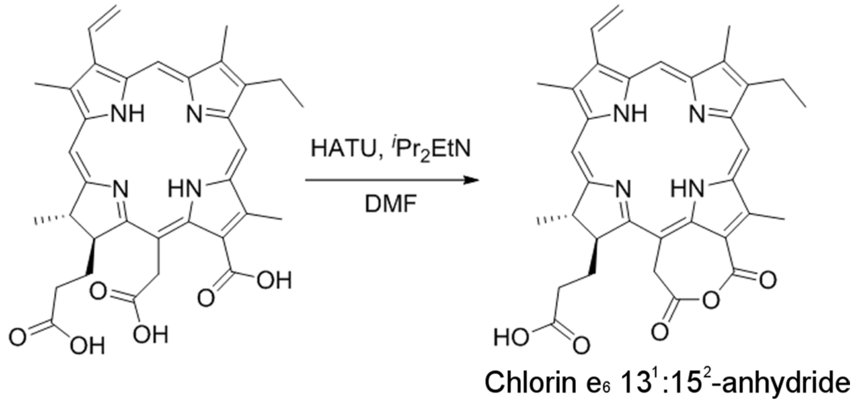


**Supplementary Figure 2: Ce6 pre-activated to form an anhydride ring between two carboxyl groups.** See methods for details of synthesis.

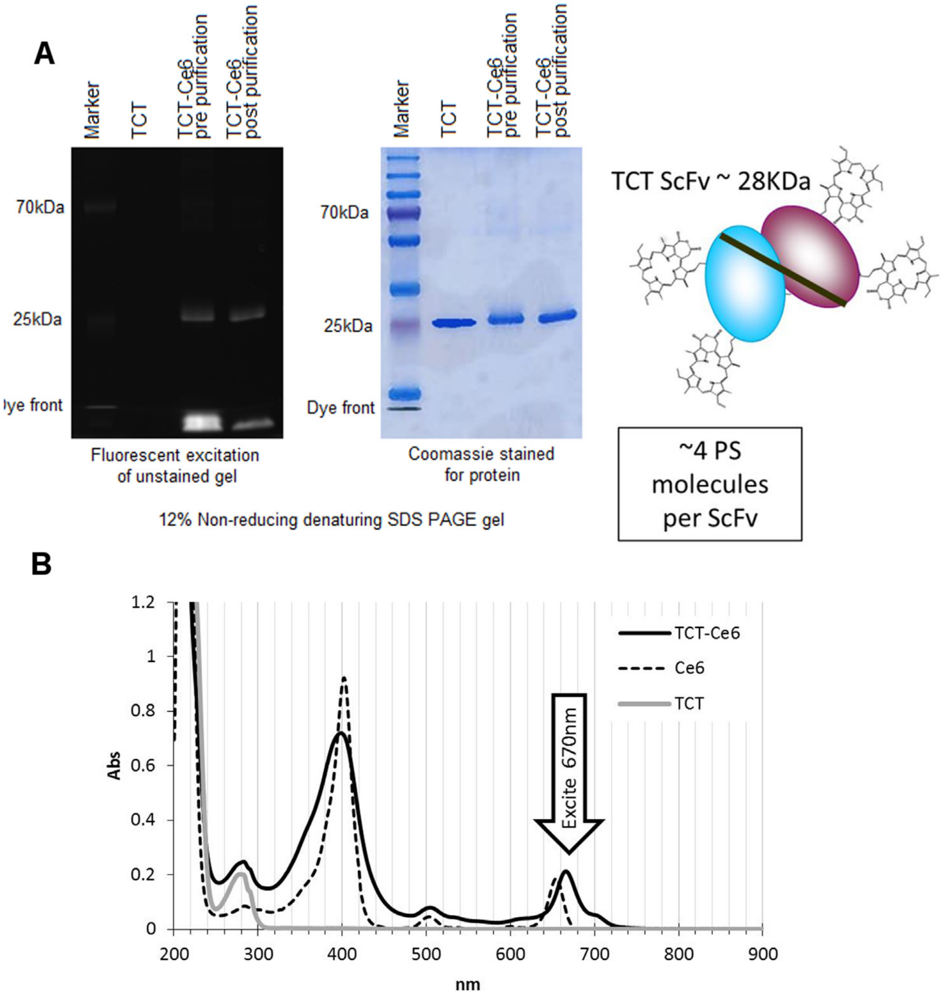


**Supplementary Figure 3: Analysis of purity and photo-physical spectral properties of TCT-Ce6.** (**A**) Samples were taken throughout the conjugation and purification process and ran on non-reducing SDS-PAGE. Gels were Coomassie stained for protein after being imaged for PS fluorescence. Almost all unconjugated dye (seen at the dye front with the 10 kDa marker) was removed from the final product, conjugated dye was shown to be covalently bound (seen at the height of the protein). The small amount of non-covalent material left in the conjugate was tightly bound and could not be removed with the addition of excipients during the purification process. (**B**) TCT-Ce6 was analysed by UV-Vis spectroscopy shown overlayed with a spectrum of free Ce6 (dashed) and free TCT (grey). The Ce6 peak at 400nm broadened and the 660 nm peak red-shifted upon conjugation. Despite this the extinction coefficient of the free dye at 400nm was used to determine the dye concentration of the conjugate, protein concentration was determined by Bradford assay.

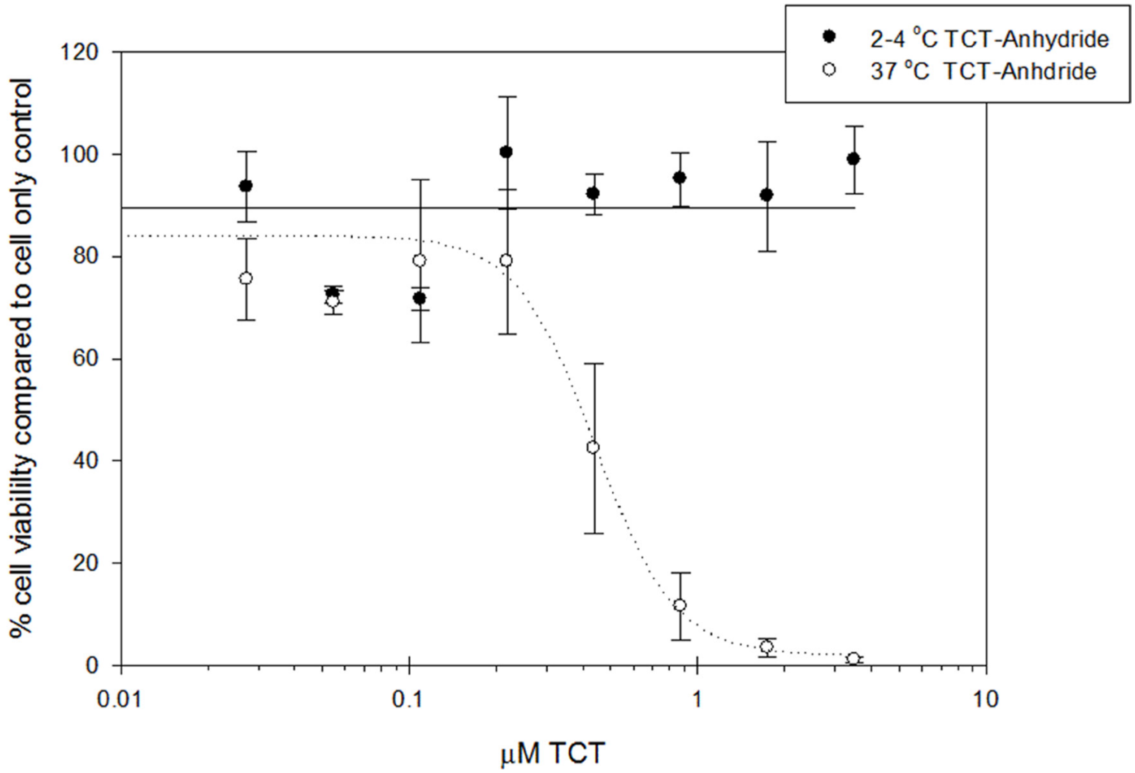


**Supplementary Figure 4: Cell uptake of TCT-Ce6 is prevented at low temperatures.** Dose dependent PDT based cytotoxicity of TCT-Ce6 on HER2 positive oesophageal cells. When incubated at 2–4° C during cell uptake of TCT-Ce6 PDT results in no cytotoxicity when cells are returned to 37° C supporting the hypothesis TCT-Ce6 is taken up by receptor driven endocytosis. PDT treatment; cells were exposed to various concentrations of the drug over one hour at either 37° C or 2–4° C, cells were then washed twice, and returned to warm media prior to exposing cells to a 670 nm laser. Cell viability was measured 24 hours later *via* MTT assay (See method in main paper for more detail).

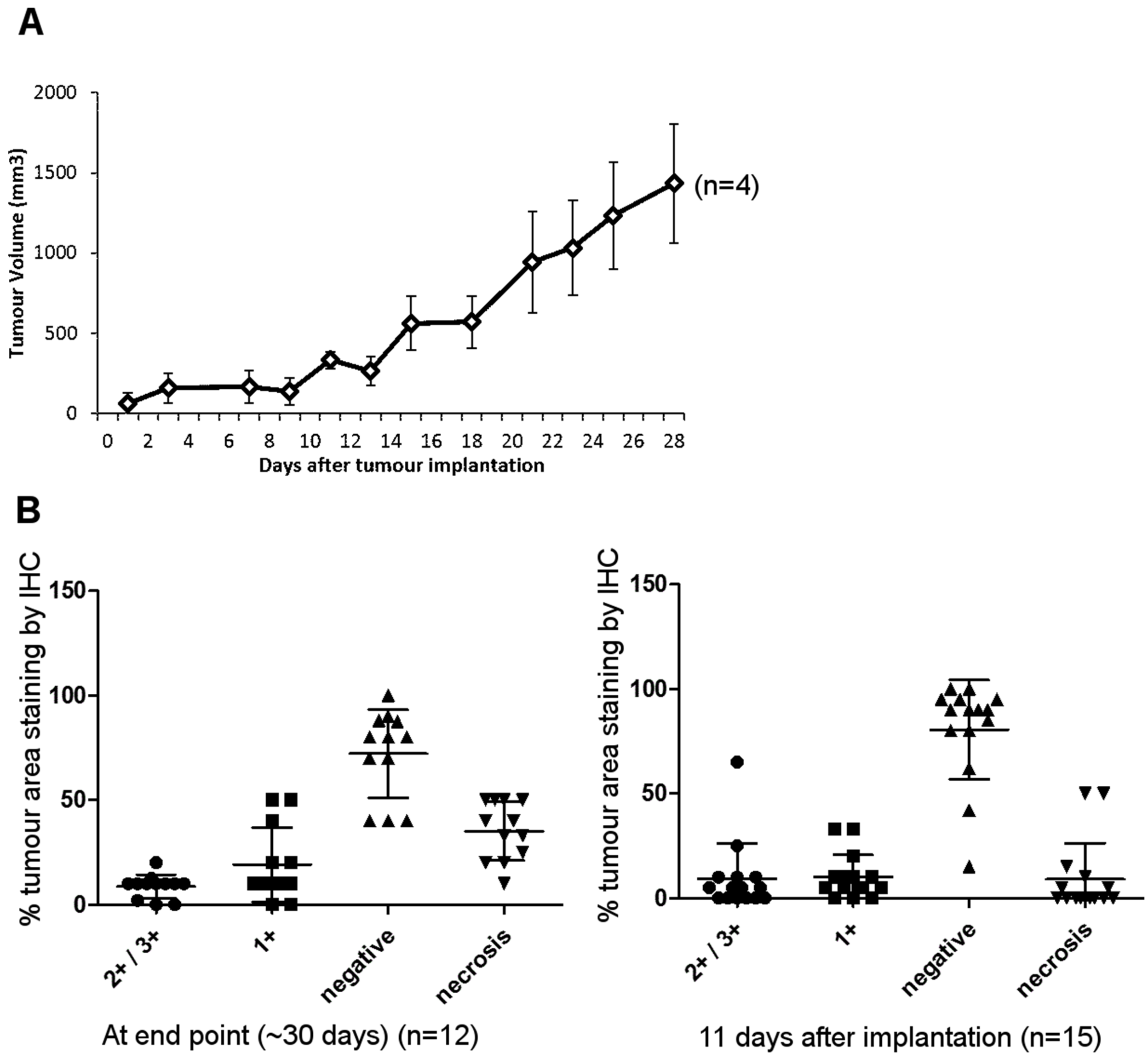


| | | Average | SD | |
|---|---|---|---|---|
| End Point tumours | 2+ / 3+ HER2 positive | 8.7 | 5.6 | 100% |
| | 1+ HER2 positive | 19.2 | 17.8 | |
| | HER2 negative | 72.1 | 21.1 | |
| | Necrosis | 35.1 | 13.9 | |

| | | Average | SD | |
|---|---|---|---|---|
| Day 11 tumours | 2+ / 3+ HER2 positive | 9.3 | 16.8 | 100% |
| | 1+ HER2 positive | 10.1 | 10.6 | |
| | HER2 negative | 80.6 | 23.7 | |
| | Necrosis | 9.0 | 17.2 | |

**Supplementary Figure 5: Characterisation of a mouse flank xenograft model of HER2 positive human OA.** (**A**) The tumors grew from ~day 10 until between 25–30 days after subcutaneous injection when they reached the size limit. (**B**) Tumours at day 11 (*n* = 15) and at the experimental endpoint (~day 30) ) (*n* = 12) underwent IHC investigation for HER2. Tumours were mostly HER2 negative on IHC. Bars represent Mean ± SD. Proportion of tumour area at 100% was divided into negative staining or positive staining either 1+ (low), 2+ or 3+ (high). Proportion of necrosis was separately measured as a percentage of the whole mass.

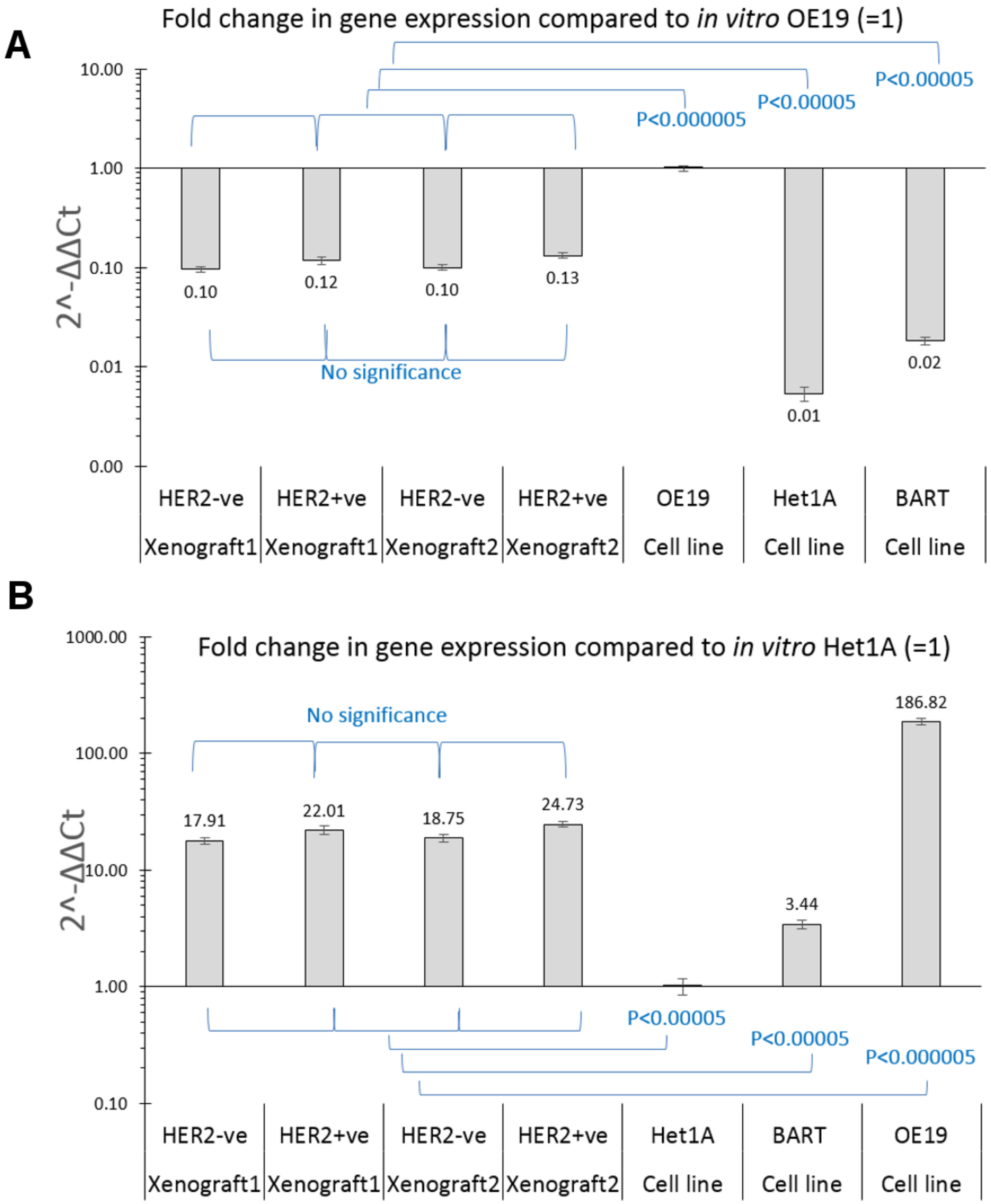


**Supplementary Figure 6: Quantification of HER2 in the xenograft tumour compared to cultured cell lines.** Two OE19 tumours were taken from the SKID mouse flank at tumour burden and the HER2 +ve and HER2 –ve areas were micro-dissected from FFPE slides under a microscope. RNA was extracted and on 200 ng quantitative PCR for HER2 mRNA and 18Sr RNA was carried out. HER2 mRNA levels were normalised to the amplification of the ribosomal 18S rRNA in each sample. The same qPCR was also carried out on RNA extracted from two esophageal cell lines; normal squamous (Het1A), and cancerous columnar (OE19). The fold change in gene expression calculated using the 2^-ΔΔCt method. Data shown as Mean and SD of technical replicates ($n = 3$). The mRNA levels of HER2 was not significantly different between the negative and positive areas of the tumours (*T*-TEST). Compared to OE19 (Cancer), all areas independently showed a ~10 fold reduction in HER2 mRNA levels (*T*-TEST $P < 0.00005$) and compared to HET1A (normal) they all showed a ~20 fold increase (*T*-TEST $P < 0.00005$).

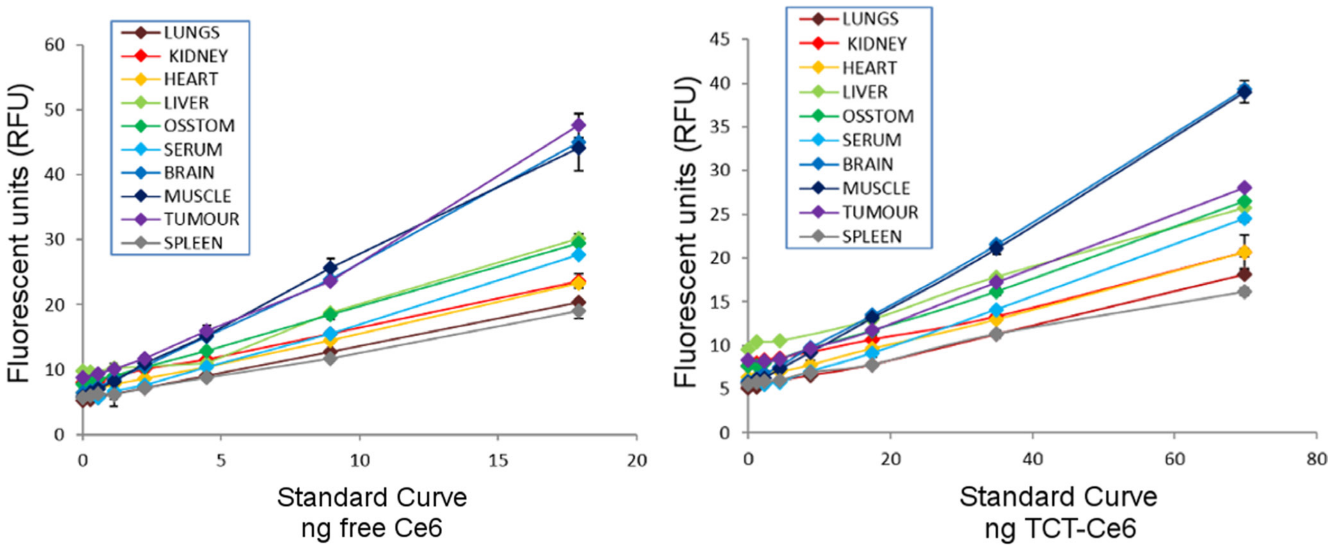


**Supplementary Figure 7: Tissue distribution of free Ce6 at various timepoints after I.V injection.** Results for Fig 4 were controlled for tissue specific auto-fluorescence and quenching with standard curves of either free or conjugated Ce6 dissolved in each tissue. Standard curves of known concentration of TCT-Ce6 or free Ce6 in Solvable™ fitted with Y = mX+C where Y = RFU, X = ng Ce6 (either free or within the conjugate), M = Gradient or quenching power and C = Y-axis intercept or autofluorescence.

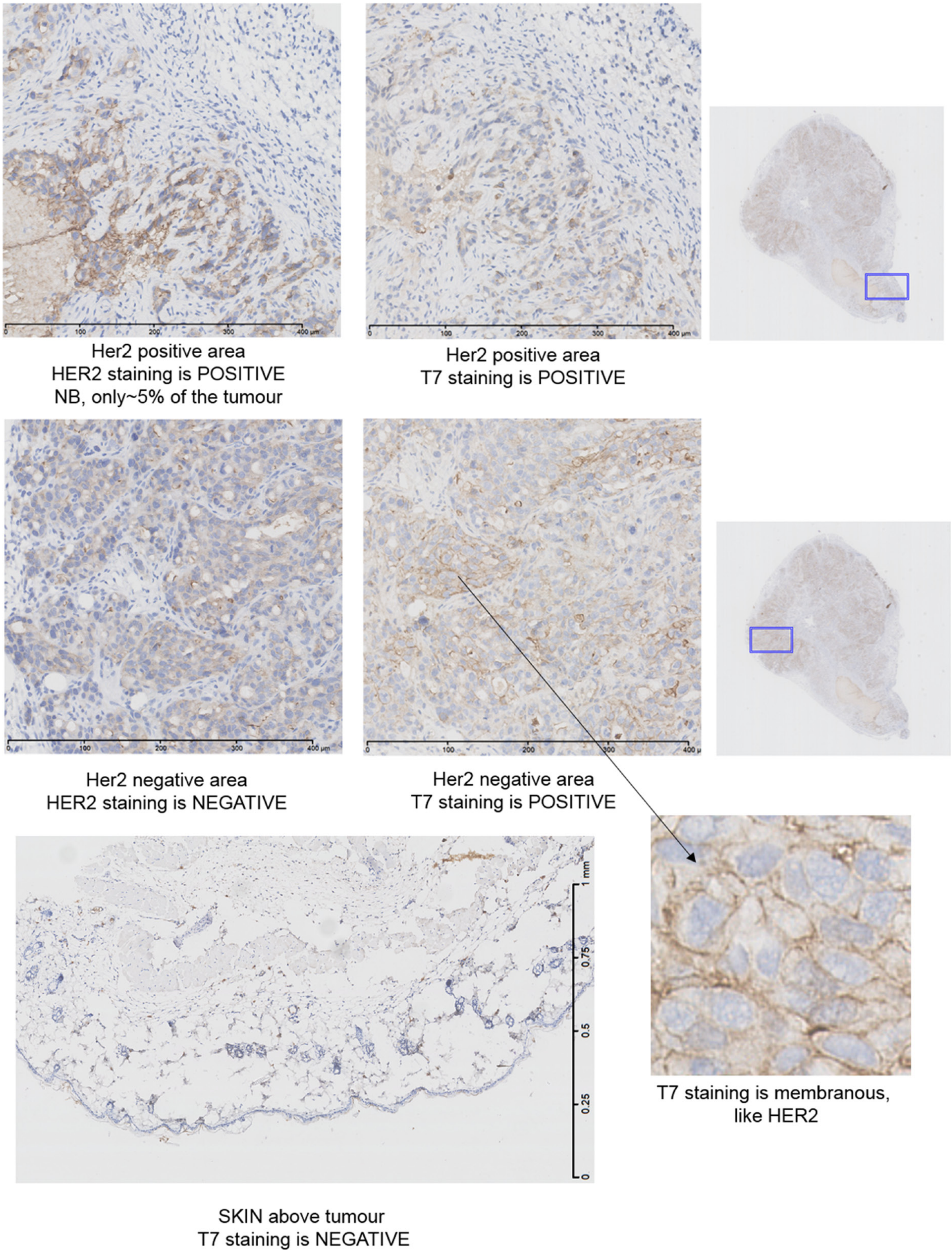


**Supplementary Figure 8: IHC distribution of the drug in the tumour and surrounding skin at 4 hours.** Example images of IHC detecting HER2 or T7 tag (part of TCT-Ce6) in FFPE tissue in a day 11 tumour taken 4 hours after TCT-Ce6 injection ($n$ = 5). The skin directly above the tumour was also taken and stained for T7 ($n$ = 3). At 4 hours TCT-Ce6 and HER2 co-localised in the same areas but TCT-Ce6 could also be seen in other areas throughout the tumour. TCT-Ce6 could be seen specifically in the tumour tissue in a membranous pattern and not in the stoma or vasculature, suggesting selective uptake. NB; at 24 hours after I.V injection ($n$ = 5) the pattern was similar but the amount of TCT-Ce6 almost negligible, there was also no TCT-Ce6 in the skin at 24 hours ($n$ = 3)). (not shown). Tumours after a saline injection were also stained and showed no T7 staining ($n$ = 3) (not shown).

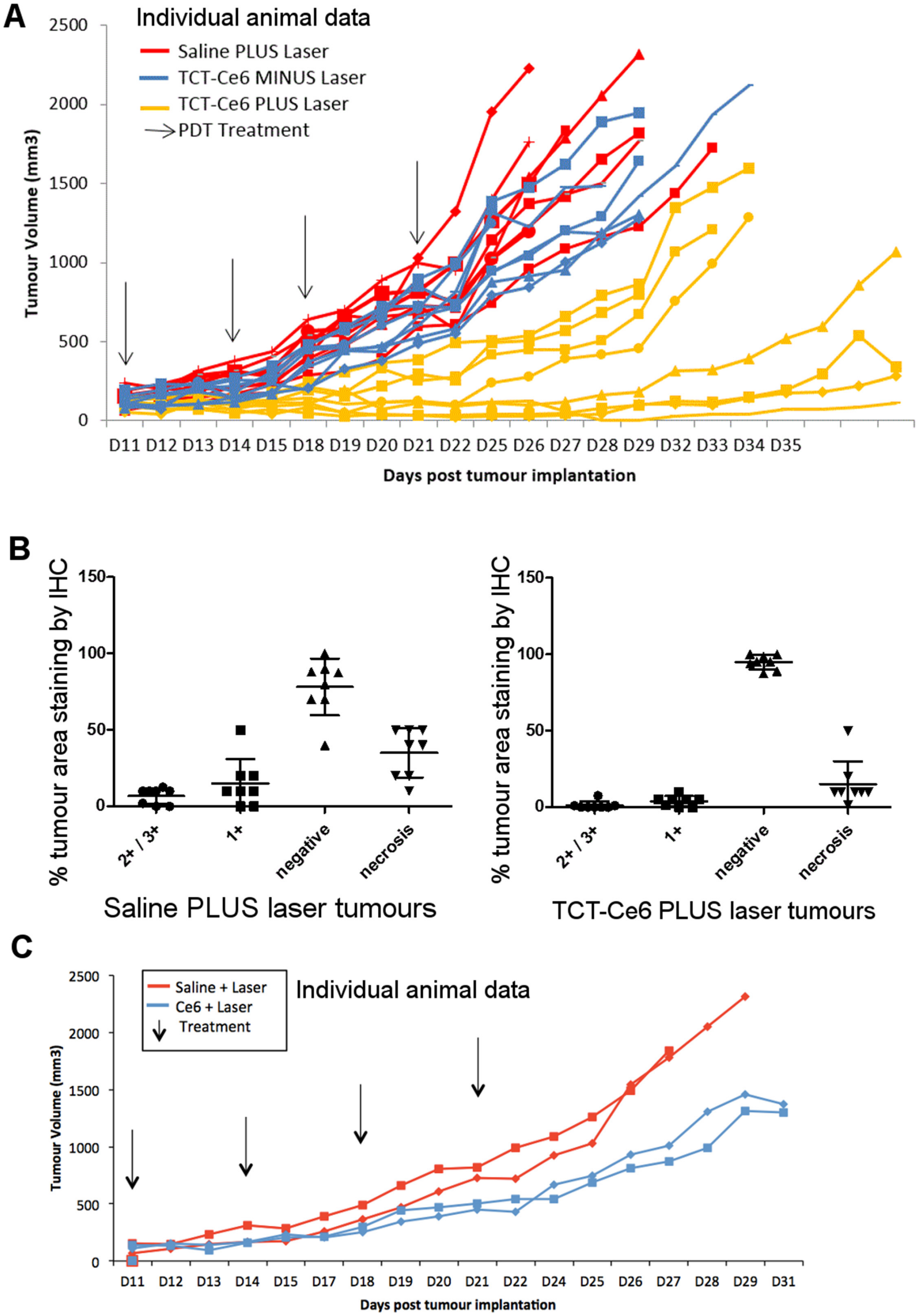


**Supplementary Figure 9: *In vivo* PDT; individual animal data, kaplan meier survival curves and tumour HER2 characterisation at endpoint.** Effect of PDT treatment using TCT-Ce6 on OE19 subcutaneous flank tumours. (**A**) Individual animal data, each group $n = 8$. Arrows represent PDT treatment time points. Tumours at the end of the experiment were taken for IHC analysis of HER2, quantification of the staining is shown (**B**), no differences in HER2 staining were seen between treated and non-treated animals. (**C**) Additional small study comparing the same treatment regimen as in A but using free Ce6, individual animal data, each group $n = 2$.
